# GRANDMOTHER INITIATIVES IN FAMILY TRANSFORMATION: OUTCOMES OF AN INTERVENTION STUDY

**DOI:** 10.1093/geroni/igae098.3377

**Published:** 2024-12-31

**Authors:** Carol Musil, Alexandra Jeanblanc, Christopher Burant, Jaclene Zauszniewski

**Affiliations:** Case Western Reserve University, Cleveland, Ohio, United States; Case Western Reserve University, Cleveland, Ohio, United States; Case Western Reserve University, Cleveland, Ohio, United States; Case Western Reserve University, Cleveland, Ohio, United States

## Abstract

To address the well-documented stress and health challenges faced by grandmothers raising grandchildren, a sample of 342 grandmothers living with grandchildren participated in a national online, NIH funded randomized clinical trial testing two methods to reduce stress. We evaluated whether the 4-week Grandmother Initiatives in Family Transformation (GIFT) intervention (Resourcefulness Training © via video plus structured journaling) compared to a 4-week unstructured journal-only condition (with video instruction) improved grandmothers’ individual and family well-being: a) mental health (general mental health and depressive symptoms), b) physical health (self-rated and general health), c) family well-being (family functioning) and d) resources (resourcefulness, support, and reward) at 2, 12, and 24 weeks post-intervention. We ran RM-ANOVA for each outcome, looking at changes over time, time by arm, time by days journaling, and time by number of video views for their arm. Grandmothers in both arms showed significant improvements on general mental health and family functioning and reduced depressive symptoms and stress across time, establishing that both arms received helpful interventions; there were no changes in physical health indicators. Participants in both arms reported improved resourcefulness, support, and caregiving reward. Significant improvements over time in mental health and support were reported by participants journaling more frequently. The number of video views was significant over time only for resourcefulness, although there were between-subjects effects for video views for general mental health and depressive symptoms. Implications for online interventions to reduce stress, and the impact of active participation (journaling, practice and video support) are discussed.

